# A curved compliant spinal bone anchor to enhance fixation strength

**DOI:** 10.1371/journal.pone.0315629

**Published:** 2024-12-19

**Authors:** Esther P. de Kater, David J. Jager, Paul Breedveld, Aimée Sakes

**Affiliations:** 1 Bio-Inspired Technology Group, Faculty of Mechanical Engineering, Department of BioMechanical Engineering, Delft University of Technology, Delft, The Netherlands; 2 Department of Electronic and Mechanical Support Division, Faculty of Electrical Engineering, Mathematics and Computer Science, Delft University of Technology, Delft, The Netherlands; Columbia University Vagelos College of Physicians and Surgeons, UNITED STATES OF AMERICA

## Abstract

Pedicle screws have long been established as the gold standard for spinal bone fixation. However, their fixation strength can be compromised in cases of low bone density, particularly in osteoporotic bone, due to the reliance on a micro-shape lock between the screw thread and the surrounding bone. To address this challenge, we propose augmenting conventional pedicles screws with a curved compliant anchor. This anchor integrates a curved super-elastic nitinol rod that is advanced through a canulated pedicle screw, forming a macro-shape lock within the vertebral body to aid the fixation strength. Both placement safety and fixation strength of this novel spinal bone anchor were validated on tissue phantoms (Sawbones). The radius of the curved compliant anchor’s path demonstrates high precision while exhibiting strong dependence on the bone density in which the anchor is placed. When the curved compliant anchor is combined with a conventional pedicle screw, the mean maximum pull-out force elevated to 290 N, marking a 14% enhancement in pull-out resistance compared to using pedicle screw alone. Further augmentation with multiple curved compliant anchors holds promise for even greater fixation. The application of a curved compliant spinal bone anchor offers a promising means of increasing the fixation strength of pedicles screws, which is especially relevant in challenging clinical scenarios such a patient suffering from osteoporosis.

## Introduction

### Bone anchoring

Back issues, including conditions like herniated discs, spinal deformities, and spinal instability, often necessitate spinal fusion surgery. This orthopedic intervention involves the fusion of multiple adjacent vertebrae into a single bony mass. Interbody fusion stands out as the most frequently performed type of spinal fusion surgery, with an annual occurrence of over 352.000 performed cases in the United States alone [1]. This number is expected to rise in the foreseeable future, as the prevalence of degenerative disorders rises due to an aging population and advances in medical technology, which expand the number of eligible patients [2–6]. In line with these trends, the number of lumbar fusions for patients aged over 65 increased with 138% between 2004 and 2015 in the United States [2].

**Fig 1:**
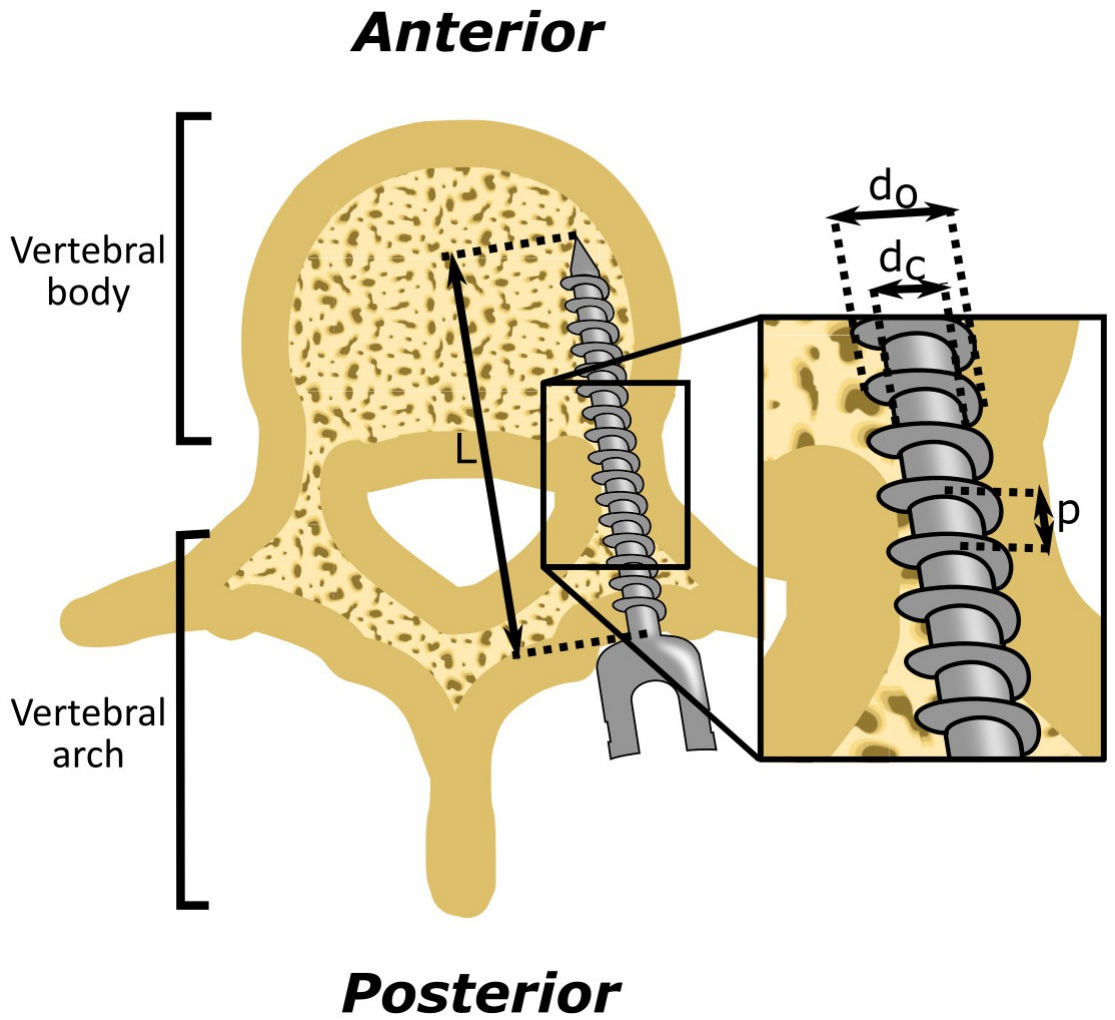
Vertebral fixation. The current golden standard uses pedicle screws with screw thread to create a fixation using micro shape-lock.

During spinal fusion surgery, the objective is to correct spinal deformities and to generate stability of the spine. In order to achieve this, a connection between adjacent vertebrae is constructed by introducing pedicle screws into the vertebrae and linking them with rods that traverse alongside the vertebrae (Fig 1). This rigid construct intends to eliminate any movement between the adjacent vertebrae, facilitating their fusion with the assistance of bone graft material. The success of spinal fusion surgery is highly dependent on the anchoring strength of the pedicle screw within the vertebra, as even minor movement between the vertebrae can hinder the desired fusion [7].

### Problem definition

While pedicle screws are considered the gold standard in spinal fusion surgery, their fixation may lack, leading to complications such as screw loosening, unsuccessful fusion, and the need for revision surgery. Understanding the vertebral anatomy is of importance in this context. Vertebrae comprise a dense and strong outer layer of cortical bone encasing porous cancellous bone. The main part of a positioned pedicle screw is surrounded by the porous cancellous bone. Only the small section of the screw within the pedicle engages with the cortical bone. Sometimes the distal tip of the screw is engaged in the anterior cortex of the vertebral body to enhance the contact area with the cortical bone. These two relatively small sections accounts for up to 85% of the screw’s pullout resistance, while the main part of the screw that is surrounded with the cancellous bone contributes only 15–20% of the pull-out resistance [8].

Pedicle screw loosening is a clinically relevant complication. Several studies have reported the prevalence, risk factors, and outcomes associated with screw loosening in spinal fusion surgery [9–12]. These complications are of particular concern in patients with poor bone quality or challenging anatomical conditions, where traditional fixation methods may be insufficient. Screw loosening is more prevalent in patient suffering from osteoporosis [7, 13]. Osteoporosis is characterized by reduced bone density due to the degeneration of cancellous bone and thinning of the cortical bone layer within the pedicle [14]. Wu et al. [12] reported a 4.7% incidence of screw loosening in non-osteoporotic vertebra, while the meta-analysis conducted by Rometsch et al. [15] revealed a significantly higher average screw loosening incidence of 22.5% in osteoporotic vertebra.

An important indicator of screw fixation strength within a homogeneous material is the Flank Overlap Area (FOA) [*mm*^2^], representing the area of material captured by the screw thread. The FOA represents the area of interaction between the screw thread and the bone, which is critical for determining the anchoring strength of the screw. The FOA can be calculated using Eq 1 [16], with *d*_*o*_ the outer diameter of the screw [mm], *d*_*c*_ the core diameter of the screw [mm], *L* the shaft length of the screw [mm], and *p* the pitch of the screw thread [mm] (Fig 1).


$$FOA=\frac{1}{4}\pi \left(d_{o}^{2}-d_{c}^{2}\right)*\frac{L}{p}$$ (1)

The magnitude of the FOA is primarily influenced by the distance between the outer diameter of the screw and the core diameter of the screw. This distance determines the area of interface between the bone and the screw thread, thereby directly impacting the anchoring effectiveness of the screw. In spinal fusion surgery, the optimization of the FOA is limited by the vertebra’s size. Pedicle screws must conform to specific dimensions of the vertebra, including the pedicle and the vertebral body, to ensure proper placement. For instance, the screw should pass through the pedicle without breaching the cortical bone layer, limiting the outer diameter, and the screw length should be selected to avoid penetrating the anterior cortical wall. While reducing the screw’s inner diameter has the potential to increase the FOA, it would come at the cost of weakening the screw itself. Similarly, reducing the screw thread pitch may enhance the FOA for screws in a homogeneous material, but this principle does not apply to pedicle screws used in porous, non-homogeneous bone. A pitch smaller than the pore size of cancellous bone would hinder effective bone material capture between the threads, compromising screw fixation strength.

While the screw thread’s micro-fixation mechanism performs well in homogeneous material such as dense cortical bone, it is not ideally suited for reliable fixation within porous cancellous bone. Achieving fixation in cancellous bone, particularly in patients suffering from osteoporosis, presents a considerable challenge due to the compromised bone quality, reducing the fixation strength of pedicle screws. This underscores the need to explore methods to establish secure fixation within cancellous bone.

### State-of-the-Art: Anchoring in cancellous bone

To increase the fixation strength of pedicle screws within the cancellous bone, ongoing research explores alternative anchoring methods [17]. Cement-augmented pedicle screws are frequently employed to enhance fixation strength in osteoporotic bone. These screws feature a central lumen through which cement, such as PolyMethylMethAcrylate (PMMA), is injected. The cement exits the screw via side channels at the distal tip, flowing into cancellous bone pores to create a firm shape lock. Consequently, load on the screw transfers to the vertebral bone via the cement, nearly doubling the pull-out strength as compared to conventional pedicle screws [7]. However, there are challenges associated with the use of cement-augmented screws. Heat generated during cement hardening can result in bone necrosis [18]. Additionally, cement leakage from the vertebra via cortical defects or veins may occur, and although this rarely results in clinical complications such as Pulmonary Embolisms (PE), the occurrence of asymptomatic cement leakage is high [19–21]. While the rates of asymptomatic cement leakage and asymptomatic PE are relatively high, it is important to note that the occurrence of clinical consequences is rare. This distinction underscores the generally low clinical risk associated with these findings despite their frequency. In the study of Mueller et al. [19], asymptomatic perivertebral cement leakage was observed in 93.6% of patients (88 patients) and 73.3% of augmented vertebrae (165 vertebrae), most frequently occurring in the perivertebral venous system, while clinically asymptomatic pulmonary cement embolism was noted in 4.1% of patients (4 patients). Martín-Fernández et al. [20] found that cement leakage was seen in 62.3% of vertebrae (650 vertebrae) with no major clinical complications, though 0.6% of patients (2 patients) experienced radicular pain, and 4.1% of patients (13 patients) developed deep infections requiring surgical debridement. However, it is important to note that the removal of screws, whether for the management of infection or for revision procedures (such as in cases of pseudarthrosis or adjacent segment disease), can be significantly complicated by the presence of cement augmentation. The cement’s integration with the bone and its role in providing additional stability make these procedures more challenging and may increase the risk of damage to the surrounding bone and tissue [7, 20].

**Fig 2:**
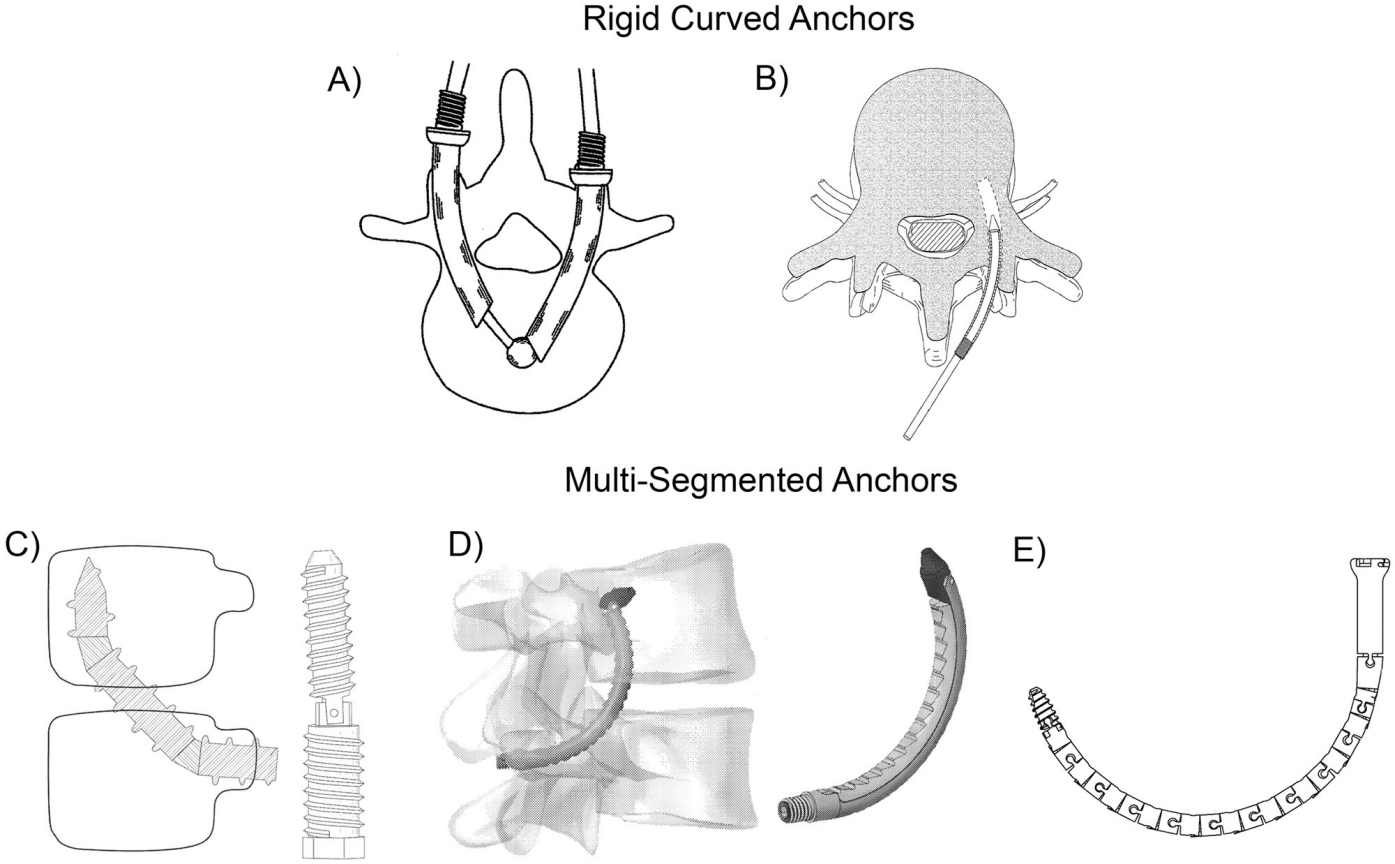
Alternative anchoring methods. Curved anchors are proposed in the patent literature. Rigid curved anchors consist of rigid pre-bend elements and are proposed by A) Ben-Arye et al. [23] and B) Matityahu et al. [24]. Multi-segmented anchors contain multiple segments that can move with respect to each other and are proposed by C) Aghayev *et al*. [25], D) Glerum *et al*. [26] and E) Meek *et al*. [27].

Another alternative anchoring method, outlined in patent literature but not yet clinically implemented, involves the utilization of curved anchors [22], see Fig 2. The curved path of such an anchor could enhance fixation strength by employing a macro shape-lock between the spinal bone anchor and the cancellous bone in the vertebral body. Rigid curved anchors, described by Ben-Arye *et al*. [23] and Matityahu *et al*. [24], can be placed in a pre-drilled curved tunnel. Unlike conventional pedicle screws, these anchors rely solely on macro-fixation within the vertebral body, omitting screw threads and thus the associated fixation strength. The patents by Aghayev *et al*. [25], Glerum *et al*. [26] and Meek *et al*. [27] propose anchors composed of separate segments capable of bending with respect to each other to create a curved macro-shape fixation. Utilizing conventional joints or helical shapes with interlocking teeth facilitates the desired compliance of the anchor while maintaining segment connection. These compliant anchors assist in placement within the vertebra, and potentially enable the use of a macro-shape fixation in combination with the conventional pedicle screws. However, the use of joints weakens the anchor structure, posing a risk of implant failure under high loads acting on the bone anchors.

### Goal of this study

The objective of this study is to develop and evaluate a novel curved compliant bone anchor, intended as an additional means of fixation, that supplements the conventional pedicle screw and enhances fixation strength by establishing a macro-shape lock within the vertebral body. Our aim is to achieve this macro-shape lock using a compliant structure as to avoid the use of joints that may weaken the anchor.

## Anchor development

### Proposed solution

**Fig 3:**
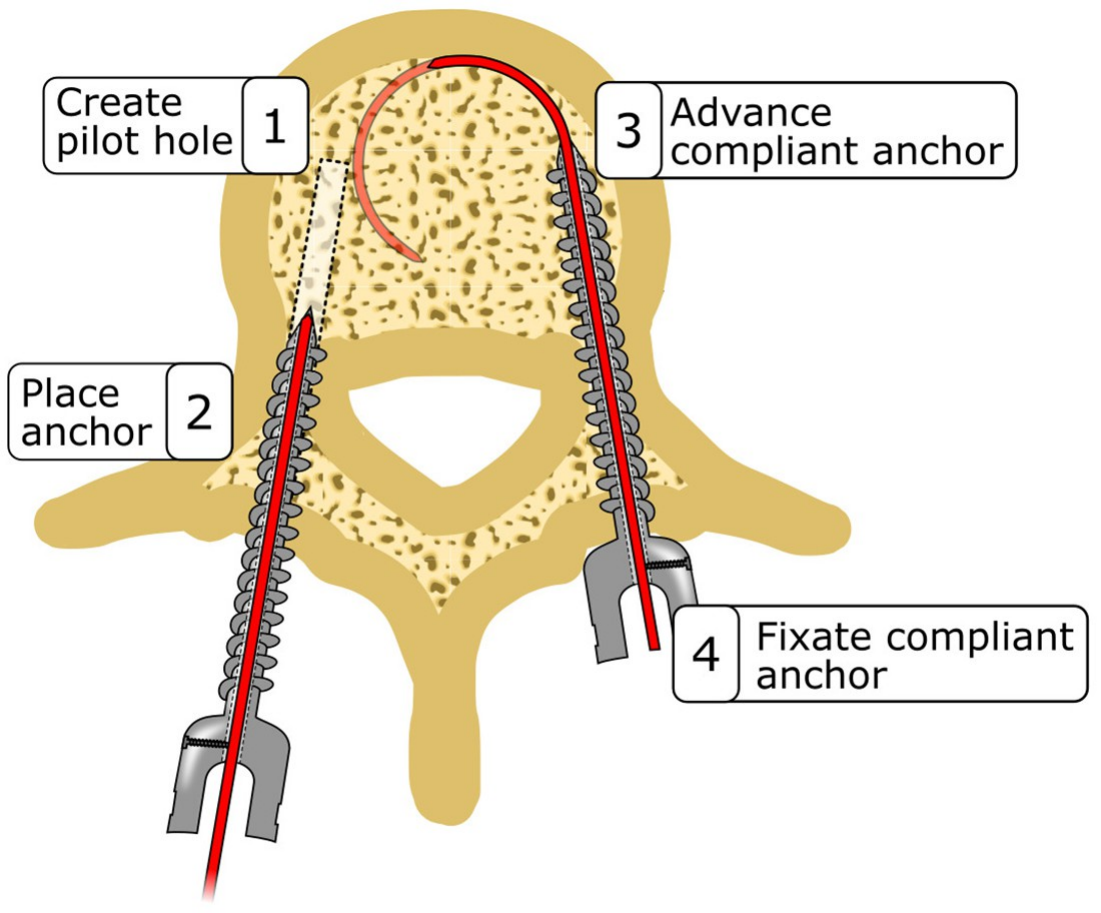
The curved compliant anchor combined with the currently used pedicle screws is placed in four steps: 1) Create the pilot hole, 2) place the anchor, 3) advance the curved compliant anchor and 4) fixate the curved compliant anchor.

To enhance the fixation strength of conventional pedicle screws, we propose the incorporation of a curved compliant anchor that can be advanced through a canulated pedicle screw, providing and additional mode of fixation alongside the conventional screw threads. Utilizing an elastic material for the curved compliant anchor eliminates the need for intricate joints or structures, ensuring smooth advancement of the curved compliant anchor through the straight lumen of the cannulated pedicle screw (Fig 3). The curved compliant anchor features a sharp tip and is curved into a precise circular arch, facilitating its insertion through the cancellous bone.

The placement of our proposed curved compliant anchor similar to conventional pedicle screws, with two additional steps after pedicle screw placement. Initially, the entry point is determined, and a cortical wall opening is created. Subsequently, a straight tunnel is formed through the pedicle into the vertebral body. The cannulated pedicle screw containing the curved compliant anchor is screwed into the straight tunnel similarly to the placement of a conventional pedicle screw. Subsequently, the curved compliant anchor is advanced through cannulated screw into the vertebral body, assuming its curved shape when exiting the cannulated pedicle screw. Once fully inserted into the vertebral body, the curved compliant anchor is secured to the pedicle screw using a bolt. This ensures that pulling forces exerted on the anchor are transferred to the bone through both the screw thread and the compliant anchor.

### Anchor design

**Fig 4:**
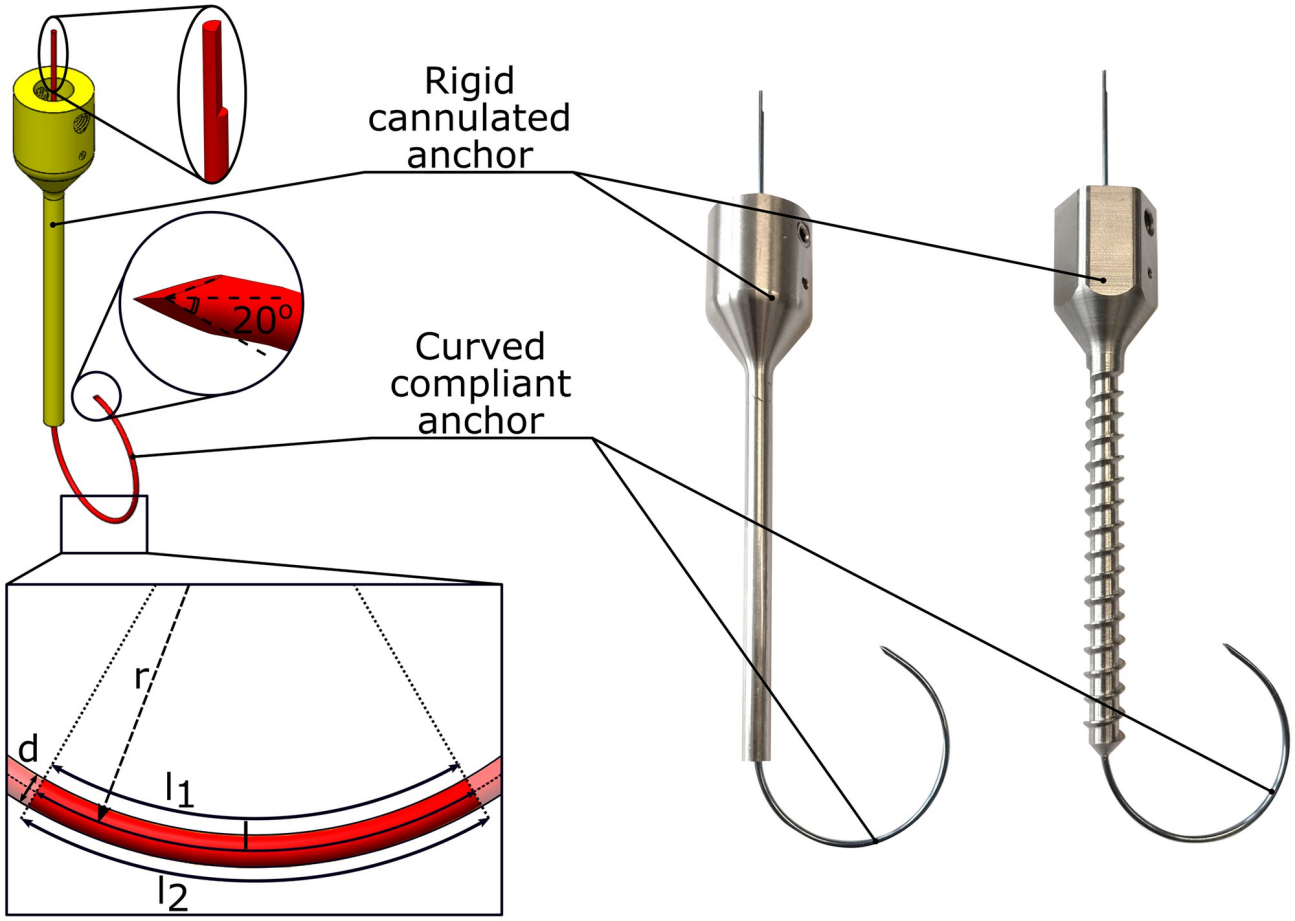
Anchor design. The spinal bone anchor comprises a rigid cannulated anchor (yellow) through which the curved compliant anchor (red) can be advanced.

The curved compliant anchor is intended to complement currently clinically available pedicle screws. Therefore, the placement and fixation strength of the curved compliant anchor will be evaluated without and with considering the fixation strength of the pedicle screw thread. For this purpose, two rigid cannulated anchors were developed: one anchor without a screw thread, and one with a screw thread, both containing a central lumen (∅1.5 mm) for the insertion of the curved compliant anchor (Fig 4). Given that advancing the curved complaint anchor through the straight lumen of the rigid cannulated anchor requires significant deformation of the curved compliant anchor, it demands the use of an elastic material to prevent plastic deformation.

Nitinol is renowned for its high elastic elongation of up to 8% without undergoing plastic deformation [28]. Additionally, nitinol is biocompatible which makes it suitable to use for long-term implementation, which is required for spinal fusion surgery. Therefore, nitinol presents an ideal material choice for the curved compliant anchor, allowing for the necessary large deformations without compromising its integrity.

The minimal radius of the nitinol rod that can be straightened without experiencing plastic deformation can be computed using Eq 2, with *R* [mm] the minimal bending radius, *d* [mm] the diameter of the nitinol rod and *ε* [–] the allowable strain of the nitinol rod [28].


$$R=\frac{d}{2*\varepsilon }+\frac{d}{2}$$ (2)

Given that cannulated pedicle screws typically accommodate Kirschner-wires (K-wires) with a diameter of up to 1.5 mm, the curved compliant anchor is designed with a similar diameter of 1.25 mm. Utilizing a super elastic nitinol rod with a diameter of 1.25 mm and an allowable strain of 0.08, yields in a minimal bending radius of approximately 8.5 mm. It was decided to use a nitinol rod with a pre-curved radius of 15 mm as this not only prevents plastic deformation but also reduces the force required to straighten the rod, while ensuring the curve fits within the vertebral body.

### Anchor prototyping

The rigid cannulated anchors were manufactured from stainless steel using conventional machining techniques. The curved compliant anchor was made of a super elastic nitinol rod (Nitinol Device and Components, 1.25mm, mechanically polished). To achieve the desired curvature, the nitinol rod was placed in a mold to ensure the desired curvature and subsequently heated for a minimum of 20 minutes at 500°C. To facilitate smooth advancement of the curved compliant anchor through bone, the nitinol tip was sharpened with a bevel angle of 20°, similar to the tips of currently used K-wires [29, 30]. At the opposite end of the curved compliant anchor a flattened section was incorporated to enable the connection to a linear stage for controlled placement and alignment of the curvature of the compliant anchor within the vertebra.

The curved compliant anchor can, after advancement through the rigid cannulated anchor, be secured to the rigid cannulated anchor using a small bolt. The design of the rigid cannulated anchor is such that it can be effortlessly connected with two bolts to a pull-out testing machine especially designed to validate the fixation strength of the anchor.

## Anchor placement experiment

### Experimental goal

Accurate placement of a spinal bone anchor is crucial to prevent damage to surrounding anatomy. The compliant nature of the curved compliant anchor makes that the interaction forces between the cancellous bone and the curved compliant anchor could influence the anchor path during insertion. A higher bone density increases these interaction forces during advancement of the curved compliant anchor. Consequently, these forces may result in a deviation from the intended path. The aim of the Anchor Placement Experiment was therefore to investigate whether and how bone density affects the accuracy of placement of the curved compliant anchor.

### Experimental variables

The independent variable in this experiment was the bone density. The effect of bone density on the placement accuracy of the curved compliant anchor was investigated by inserting the curved compliant anchor into three types of bone phantoms (SawBones) with compressive strengths of 5, 10 and 15 Pounds per Cubic Food (PCF), representing severe osteoporotic, osteoporotic and healthy bone, respectively [31].

The dependent variable was the anchor path radius. The radius of the circular path followed by the curved compliant anchor was measured and compared with the radius of the curved compliant anchor in free space (15 mm).

The controlled variables in the experiment were the introduction velocity (1 mm/s) of the curved compliant anchor and the radius and length of the curved compliant anchor in free space (radius: 15 mm, length: 70 mm), which remain constant. Only the anchor without screw thread was tested in this experiment.

### Experimental facility

**Fig 5:**
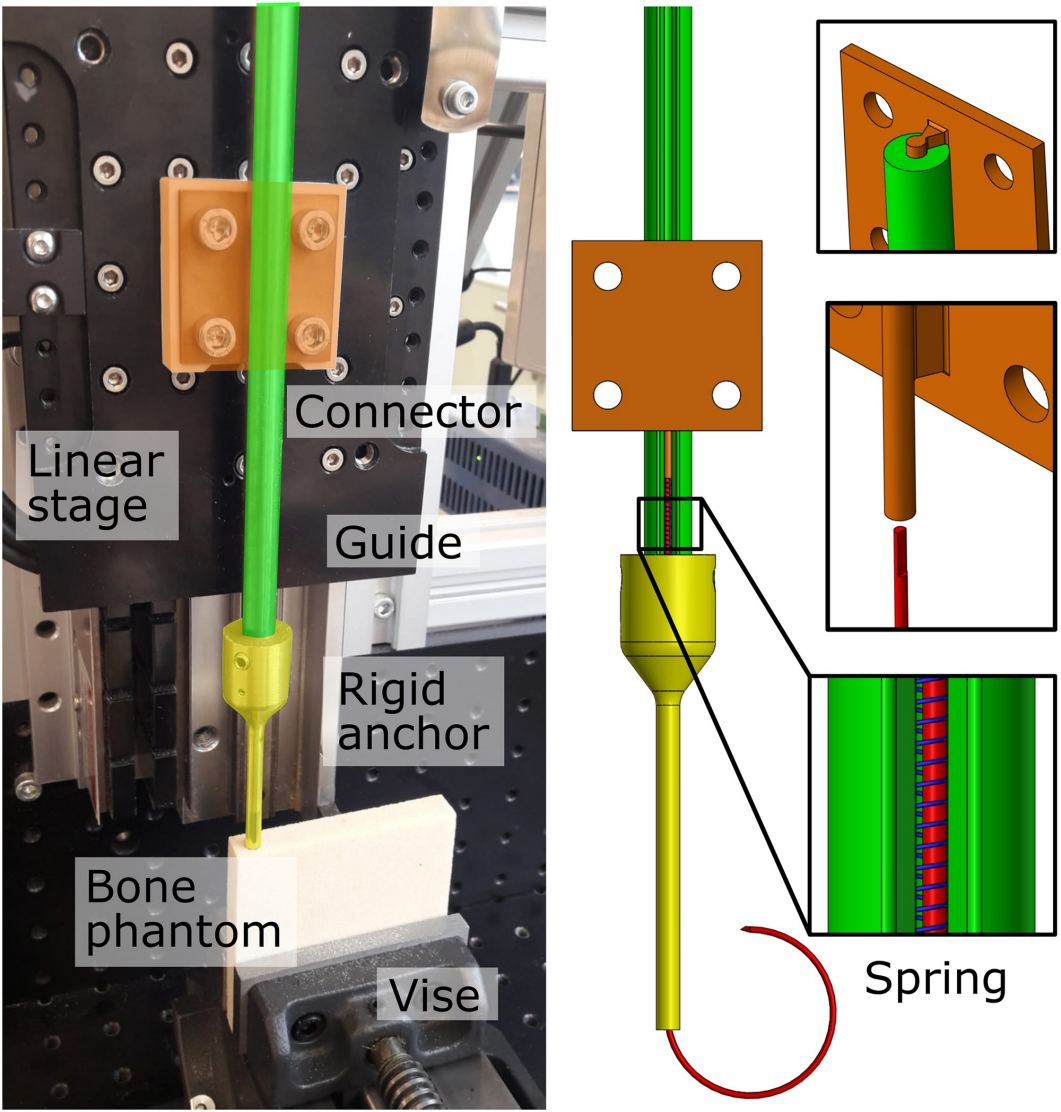
Experimental facility placement validation. A linear stage is used to advance the curved compliant anchor (red) through the non-threaded rigid cannulated anchor (yellow) and the bone phantom via the connector (orange). Buckling of the compliant anchor is prevented by the rigid guide (green) and a compression spring (blue).

The experimental facility is depicted in Fig 5. The curved compliant anchor (red, nitinol), housed within the non-threaded rigid cannulated anchor (yellow, stainless steel), is connected to the linear stage using a connector (orange, brass). The connector anchor ensures accurate placement of the curved compliant anchor within the bone phantom (10 mm x 70 mm x 70 mm). To prevent buckling of the curved compliant anchor during advancement through the bone phantom, a rigid guide (green) and a spring (blue, Verenfabriek De Spiraal, ∅_*outer*_ = 2 mm, ∅_*w*_ = 0.2 mm) were positioned around the proximal part of the curved compliant anchor.

### Experimental protocol

Bone phantoms were prepared with a bandsaw, after which a cylindrical hole (Ø3 mm, length: 30 mm) was created using a drill press. The curved compliant anchor was inserted into the rigid cannulated anchor without screw thread, ensuring alignment of the distal tips of the concentric anchors. Subsequently, the concentric anchors were placed into the pre-made hole of the bone phantom. The spring was then positioned around the curved compliant anchor and the compliant anchor was attached to the linear stage via the connector. Subsequently, the rigid guide was pushed over the connector, the compliant anchor, and the spring.

At this stage, the linear stage was employed to advance the curved compliant anchor through the bone at a velocity of 1 mm/s over a distance of 70 mm, corresponding to the length of the curved section of the anchor (70 mm). Upon completion of the anchor placement, the bone phantom was filed down until the curved compliant anchor became visible. Subsequently, the curved compliant anchor was removed, leaving the tunnel through which the anchor passed visible for analysis. This test was performed in three times in each bone density (5 PCF, 10 PCF and 15 PCF).

### Data analysis

After removal of the curved compliant anchors, the bone phantoms were scanned using a document scanner. Subsequently the scanned images were analyzed using Matlab R2019b. Initially, a reference measurement was taken to establish the scale of the image accurately. Subsequently, the radius of the curved compliant anchor’s path was determined by selecting 10 points alongside the circular arch of the anchor path with a mouse click. The radius of the curved compliant anchor was then constructed using the least square error method.

### Anchor placement results

**Fig 6:**
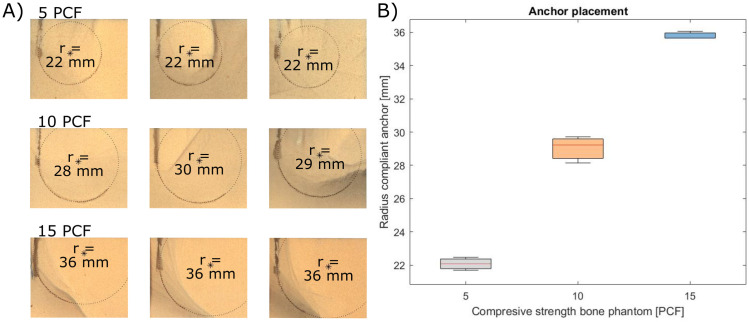
Results placement validation. (A) Cut through of the bone phantom showing the path of the curved compliant anchor and the corresponding radius. (B) Boxplot showing the radius of the curved compliant anchor for 5, 10 and 15 Pound per Cubic Foot (PCF) bone phantom.

The results of the experiment are presented in Fig 6. Fig 6A displays the radius of the curved compliant anchor in bone phantom with a compressive strength of 5, 10 and 15 PCF, respectively. The radius of the complaint anchor was 15 mm in free space, 22.1 mm (SD ± 0.4 mm) in 5 PCF bone phantom, 29.0 mm (SD ± 0.8 mm) in 10 PCF bone phantom, and 35.8 mm (SD ± 0.2 mm) in 15 PCF bone phantom material. Fig 6B illustrates a boxplot of the radii of the curved compliant anchor in bone phantom material with a compressive strength of 5, 10, and 15 PCF. Due to the low number of repetitions, no formal statistical tests were performed. The purpose of these initial experiments was to provide a preliminary evaluation on the precision of the curved compliant anchor path in bone phantoms with different densities.

## Anchor Fixation Experiment

### Experimental goal

The objective of the Anchor Fixation Experiment was to investigate the fixation strength of the curved compliant anchor in bone phantom material. The preliminary aim was to assess the impact on pull-out resistance of the curved compliant anchor without the use of a screw thread. Subsequently, the impact on pull-out resistance of the curved compliant anchor in conjunction with a rigid cannulated anchor with screw thread was compared to the pull-out resistance of a stand-alone rigid cannulated anchor with screw thread.

### Experimental variables

The independent variable in the Anchor Fixation Experiment was the anchor type. Pull-out tests were conducted using three different anchor configurations: 1) the curved compliant anchor with the non-threaded rigid cannulated anchor, 2) a stand-alone threaded rigid cannulated anchor, and 3) the curved compliant anchor in conjunction with the threaded rigid cannulated anchor.

The dependent variables were the initial pull-out force and the maximal pull-out force. The pull-out force is defined as the axial force exerted on the anchor and was measured using a force sensor (Futek, LCM300, 4448 N). The initial pull-out force refers to the amount of force required to begin the dislodgement or loosening of the screw from the bone, while the maximum pull-out force is the highest force needed to remove the screw entirely once loosening has commenced. Clinically, the initial pull-out force is the more relevant parameter, as it represents the threshold at which screw loosening starts, which is the primary concern in maintaining the stability of the fixation. Understanding this distinction helps to focus on the factors that affect initial loosening, which is critical for ensuring successful outcomes in bone fixation procedures.

The controlled variable were the bone density (10 PCF, Sawbones), the pull-out velocity (3.5 mm/min), and the radius and length of the complaint anchor (radius: 15 mm, length: 70 mm). These variables remained constant throughout the experiment.

### Experimental facility

**Fig 7:**
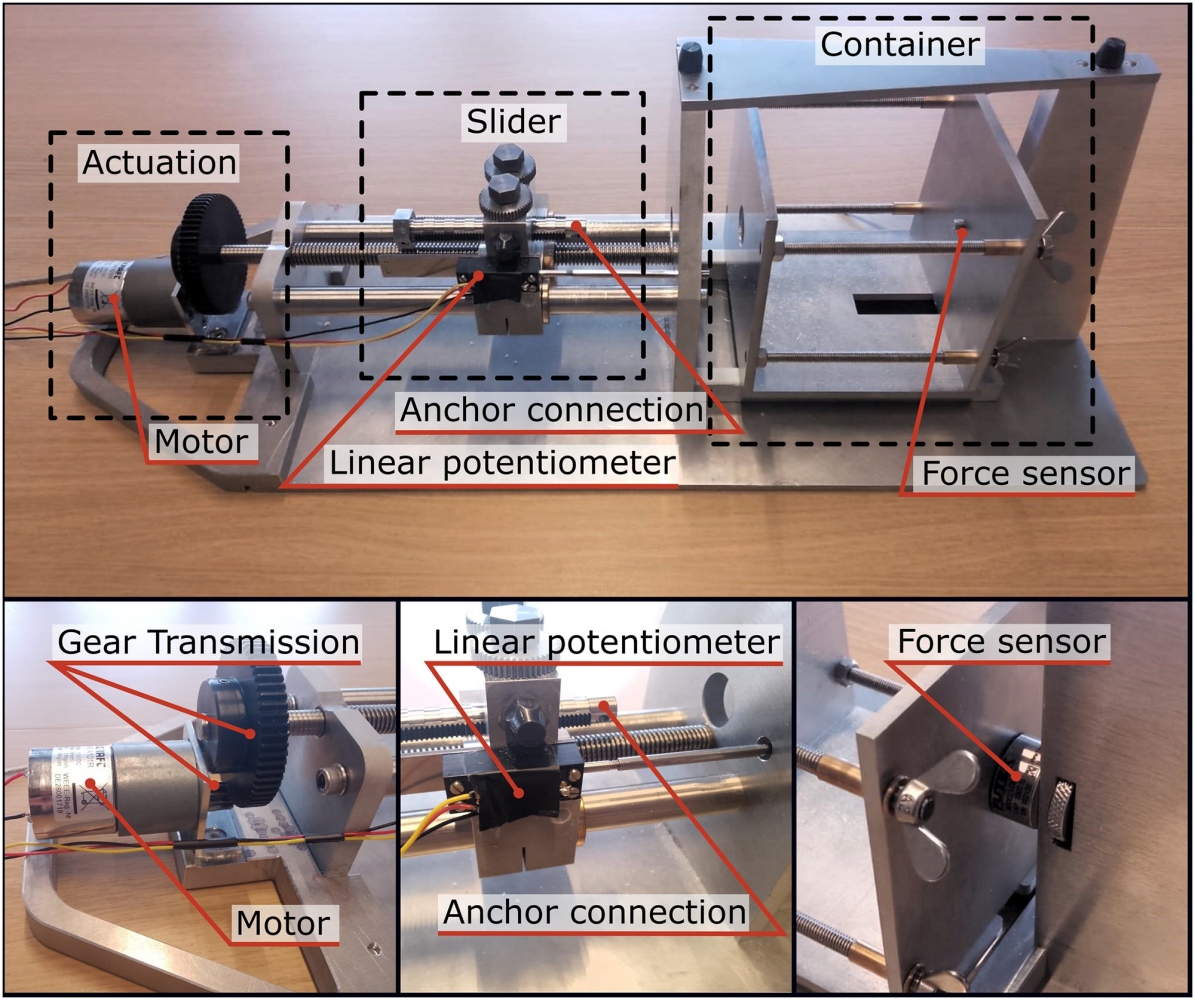
Experimental facility anchor fixation validation including the actuation that pulls the anchor via the slider out of the bone that is placed within the container. The pull-force and displacement of the anchor with respect to the bone is measured by the force sensor and the linear potentiometer.

The experimental facility is illustrated in Fig 7. The facility allows for controlled extraction of the anchor from the bone phantom while continuously measuring the pull-out force using a force sensor (Futek, LCM300, 4448 N). The initial and maximum pull-out forces were measured using this mechanical experimental facility that simulated the *in-vitro* conditions for pedicle screw fixation. For the pull-out experiment, the bone phantom containing the pedicle screws, with and without the curved compliant anchors, was positioned within the container, with the proximal end of the anchor connected to the slider. The extraction of the anchor from the bone phantom was facilitated by an electric motor (Modelcraft, RB 35, 1:600) equipped with a gear transmission (9:1), resulting in a translation speed of the slider of 3.5 mm/min. Subsequently, the screws were subjected to a continuous axial pull-out load at this constant displacement rate. Additionally, to determine the force required to initiate the extraction of the anchor, the facility was used to measure the relative displacement of the anchor with respect to bone using a linear potentiometer (Althen, 13FLP12A). The initial pull-out force was defined as the load corresponding to the initial relative displacement of the anchor with respect to the bone phantom. The maximum pull-out force was defined as the highest recorded force just before failure, characterized by a sudden drop in resistance or displacement of the screw.

### Experimental protocol

Bone phantoms (20 mm x 60 mm x 60 mm) were prepared with a bandsaw, after which a cylindrical hole (Ø3mm, length: 30 mm) was created using a drill press. The bone phantoms were composed of material with a compressive strength of 10 PCF, representing osteoporotic bone. The rigid cannulated anchor was inserted into the bone phantom using the same method as employed in the Anchor Placement Experiment. A linear stage was utilized to advance the curved complaint anchor through the rigid cannulated anchor into the bone phantom at a velocity of 1 mm/s.

Following the placement of the curved compliant anchor in the bone phantom, the curved compliant anchor was secured to the rigid cannulated anchor using a bolt (M 0.9). The bone phantom with the anchors was then positioned in the container of the experimental facility. The rigid cannulated anchor was connected to the slider using two bolts (M 2.5). The slider was then moved by the electric motor such that the linear potentiometer was fully retracted and in contact with the container and the shaft. The collected data were analyzed using Matlab R2019b. To ensure consistency in data interpretation, the data were normalized such that the anchors started moving at t = 0.

### Anchor fixation results

**Fig 8:**
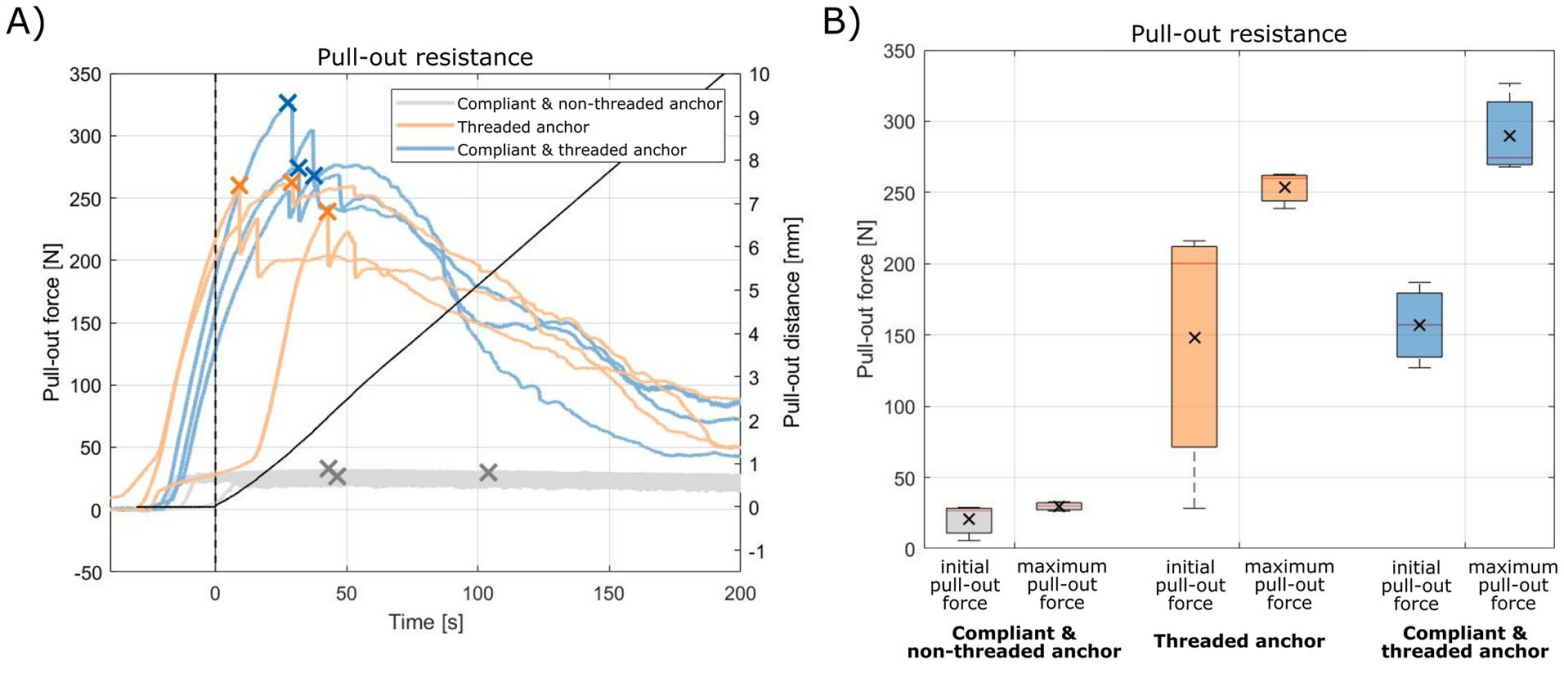
Pull-out force of the compliant anchor with the non-threaded rigid cannulated anchor, the stand-alone threaded rigid cannulated anchor, and the compliant anchor with the threaded rigid cannulated anchor. (A) Measured pull-out force of the different anchor types. Maximum pull-out force is indicated with an ‘X’. (B) Boxplot showing the maximum pull-out force of the different anchor types. The mean pull-out force for each of the anchor types is indicated with the black ‘X’.

The pull-out force of curved compliant anchor with the non-threaded rigid cannulated anchor, the stand-alone threaded rigid cannulated anchor, and the curved compliant anchor with the threaded rigid cannulated anchor is presented in Fig 8A. Fig 8B presents a boxplot representing the initial and maximum pull-out force of each anchor type. The mean initial pull-out force for the curved compliant anchor with the non-threaded rigid cannulated anchor, the stand-alone threaded rigid cannulated anchor, and the curved compliant anchor with the threaded rigid cannulated anchor is 20 N, 148 N and 157 N respectively. Additionally, the mean maximum pull-out force for the curved compliant anchor with the non-threaded rigid cannulated anchor, the stand-alone threaded rigid cannulated anchor, and the curved compliant anchor with the threaded rigid cannulated anchor is 30 N, 254 N, and 290 N respectively. The addition of a curved compliant anchor to the pedicle screw resulted in an average increase of 6% in initial pull-out resistance and a 14% increase in maximal pull-out force as compared to the stand-alone pedicle screw. Note that due to the low number of repetitions, no formal statistical tests were performed. The purpose of these initial experiments was to provide a preliminary evaluation of the potential impact, in terms of pull-out force, of the curved compliant anchor.

## Discussion

### Main findings

This study presents a novel design for a curved compliant bone anchor for use in conjunction with conventional cannulated pedicle screws. The anchor consists of a pre-curved compliant nitinol rod (∅1.25mm) with curve with a radius of 15 mm and a length of 70 mm that is advanced through a rigid canulated and threaded anchor. Placement of the combined compliant and threaded rigid cannulated anchor into the vertebra is similar to the placement of the conventional pedicle screw, with the addition of advancing the curved compliant anchor through the cannulated pedicle screw into the vertebral body after pedicle screw placement. Similarity in placement enables easy integration in the current practice of spinal fusion surgery.

Two experiments were performed to determine whether the curved compliant anchor allows for accurate placement and increases the pull-out force of current pedicle screws. In the Anchor Placement Experiment, the ability of the anchor to follow a predetermined path through cancellous bone was investigated. The radius of the complaint anchor’s path was 22 mm in a severe osteoporotic bone phantom and 36 mm in a healthy bone phantom. It was found that although the path of the curved compliant anchor is dependent on the bone density, the anchor path showed a low variability (<1mm) between samples with the same bone density. The use of pre-operative measurement of bone density, such as a Computed Tomography (CT)-scan or Dual-energy X-ray absorptiometry (DXA) [32] could be used to account for the influence of bone density of the radius of the curved compliant anchor’s path. Alternatively, real-time sensing techniques such as Diffuse Reflectance Spectroscopy (DRS), could provide feedback to alert the clinician of potential cortical breaches during placement [33].

In the second experiment, the effect of the curved compliant anchor on the pull-out resistance was investigated. The curved compliant anchor in conjunction with the threaded rigid cannulated anchor fixation strength increased by up to 14% (maximum pull-out force: 290 N) compared to the threaded rigid cannulated anchor without the curved compliant anchor. Furthermore, it was seen that the initial pull-out force was increased with 6%, which can prevent early screw loosening. The wider range of initial pull-out force in the threaded rigid anchor group may be attributed to variations in the material properties of the phantom, such as density or composition, which impact the anchor’s grip. These variations can lead to less consistent force measurements and instances where the initial pull-out force is higher without the compliant anchor.

In comparing the additional pull-out strength provided by the compliant anchor to that achieved with cement augmentation, it is important to note that while the increase (6% in initial pull-out resistance and 14% in maximum pull-out force) is modest, it represents a potentially meaningful improvement in scenarios where cement augmentation is not feasible or desired. This initial data suggests that the technology holds promise, but further refinements may be necessary to enhance its efficacy to levels that could justify broader clinical adoption. Additionally, the potential benefits of using multiple compliant anchors should be considered, as incorporating several anchors could lead to a cumulative increase in pull-out resistance, potentially making the technology more competitive with existing solutions. Thus, while the current form of the compliant anchor shows potential, further development and evaluation are needed to determine its optimal use and effectiveness in clinical practice.

### Limitations and future research

The validated curved compliant anchor is constructed from a super elastic nitinol with a diameter of 1.25 mm. A curved compliant anchor with a larger diameter is likely to increase the pull-out resistance further as this would increase the stiffness of the curved compliant anchor. However, plastic deformation of the curved compliant anchor when feeding it through the straight lumen of the rigid cannulated anchor must be avoided. This result in a maximum nitinol rod diameter of 2.2 mm using Eq 2 with an allowable strain of 0.08, and a curve radius of 15 mm. Using a larger rod diameter or a smaller curve radius also implies that a higher force is required to deform the curved compliant anchor such that it can be placed within the straight lumen of the rigid cannulated anchor and possibly that more force is required to advance the curved compliant anchor through the rigid cannulated anchor. Future research into the effects of rod diameter and curve radius on pull-out resistance seems an interesting step.

**Fig 9:**
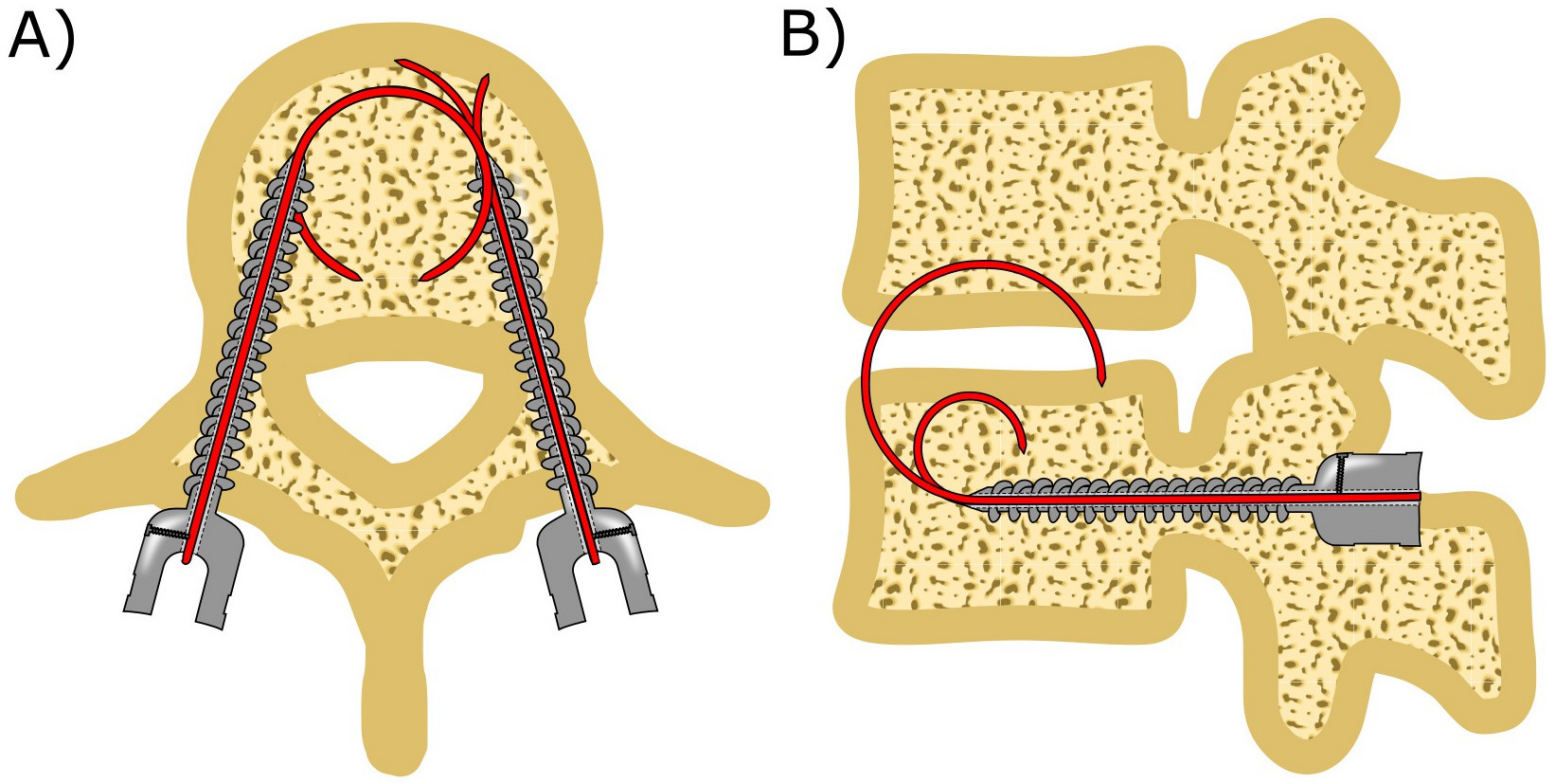
Alternative anchor designs using a compliant pre-curved anchor. (A) The use of multiple pre-curved anchors could further increase the fixation strength. (B) Anchor paths through the cortical bone layer allows for a fixation that spans multiple vertebrae.

The curved compliant anchor allows for various placement orientations, potentially increasing fixation strength by expanding contact with the vertebra (Fig 9). While our findings indicate low variability in the radius of the anchor’s path within samples of the same bone density, we acknowledge that the actual direction of this path remains unpredictable, which presents several clinical challenges. A potential concern with the use of curved compliant anchors is the risk of migration into the ventral prevertebral space, which could result in injury to surrounding tissue including nervous tissue and vascular tissue. To address these challenges, a redesign of the screw head could allow for precise control of the anchor’s insertion direction and depth. Pre-operative selection of the anchor path, considering both bone density and vertebral size, will be essential. Furthermore, the use of advanced surgical navigation systems and real-time monitoring during anchor deployment could provide continuous feedback to the surgeon, helping to avoid unintended anchor migration.

Not all vertebrae may accommodate the current anchor paths, and smaller radii should be explored to enable use in a broader range of vertebrae. Relocating the anchor’s exit point midway along the pedicle screw body could also allow for bicortical purchase, as well as offer higher versatility in anchor placement, potentially increasing pull-out strength. Nonetheless, the potential for the anchor to traverse the disc space or exit the vertebral body raises significant safety concerns, as these scenarios could cause damage to adjacent segments, interfere with interbody fusion procedures, or even result in injury to surrounding structures. While the outer cortical bone may limit the anchor’s path, further investigation is needed to ensure this assumption is valid and that the technology can be safely implemented.

While this study focused on pull-out resistance as a measure of fixation strength, future research should explore the impact of curved anchors on alternative load cases as well as cyclic load cases that may result in toggling of the anchor. Toggling resistance is crucial in preventing pedicle screw loosening as the pivoting motion of bone anchors is known to compromise the fixations strength [34]. Investigating alternative and cyclic load cases would provide a more comprehensive understanding of the anchor’s stability and resistance to loosening under various loading conditions.

The presented curved compliant anchor was designed as a spinal bone anchor in conjunction with a cannulated pedicle screw. We acknowledge that the integration of the nitinol anchor with the standard pedicle screw head does not allow for rod placement in its current form. To address this, the screw head needs to be redesigned to accommodate the nitinol anchor deeper inside the pedicle screw, which will ensure sufficient space for rod placement. This adjustment will facilitate easy integration of the anchor into the current practice of spinal fusion surgery, making it a viable option for clinical use. Additionally, the curved compliant anchor bears resemblance to the K-wires used in orthopedic procedures for fixating bone fragments. Curved compliant anchors could, therefore, potential be used as an alternative for K-wires for increased fixation strength. Besides increased fixation, curved compliant K-wires could also be used to follow the shape of more organically shaped bones, such as the pelvis, to prevent protrusion out of the bone such that damage to surrounding tissue is limited.

The experiments performed in this study were conducted using SawBones Solid Foam bone phantom material with mechanical properties similar to human bone [31]. However, its homogeneity and closed cell structure may not fully replicate the complexity of real human bone, potentially influencing anchor behavior. We acknowledge that bone density is likely to influence the performance of the curved anchors, with more osteoporotic bone potentially reducing the pull-out force, and recommend further experiments using bone phantoms with varying densities to better understand this effect. Furthermore, solely the interaction of the curved anchor within cancellous bone were investigated, neglecting the impact of the cortical bone layer. Future research should consider investigating the influence of the cortical bone layer on anchor performance. Therefore, further *ex-vivo* and *in-vivo* experiments are warranted to validate the performance of the curved anchor in more realistic bone environments. Additionally, we recognize that future studies will require a larger sample size and appropriate statistical analysis to determine the statistical significance of these findings and to validate the observed trends.

## Conclusions

The curved compliant spinal bone anchor introduced in this study featured a compliant nitinol curved rod capable of being advanced through a canulated pedicle screw. The trajectory of the curved compliant anchor proved highly predictable, even though the path is dependent on the bone density. When utilized independently, the curved compliant anchor exhibited a pull-out resistance of 30 N. Using the curved compliant anchor combined with a threaded rigid cannulated anchor, the mean pull-out resistance increased substantially to 290 N, marking a 14% enhancement in pull-out resistance compared to the threaded rigid cannulated anchor without the curved compliant anchor. Employing multiple curved compliant anchors and exploring alternative paths could further augment the pull-out resistance of pedicle screws. The curved compliant bone anchor evaluated in the present study represents a first step towards enhancing the fixation strength of bone screws in a variety of orthopedic procedures, including spinal fusion surgery.

## Supporting information

S1 Raw data Raw data of the pull-out experiments. (XLSX)

S1 Raw image Photographs of the insertion test of the compliant anchor. (PNG)
