# Supplementary figures and images for: A curved compliant spinal bone anchor to enhance fixation strength
Source: PLoS One. 2024 Dec 19;19(12):e0315629. doi: 10.1371/journal.pone.0315629 (PMC11658614; doi:10.1371/journal.pone.0315629)

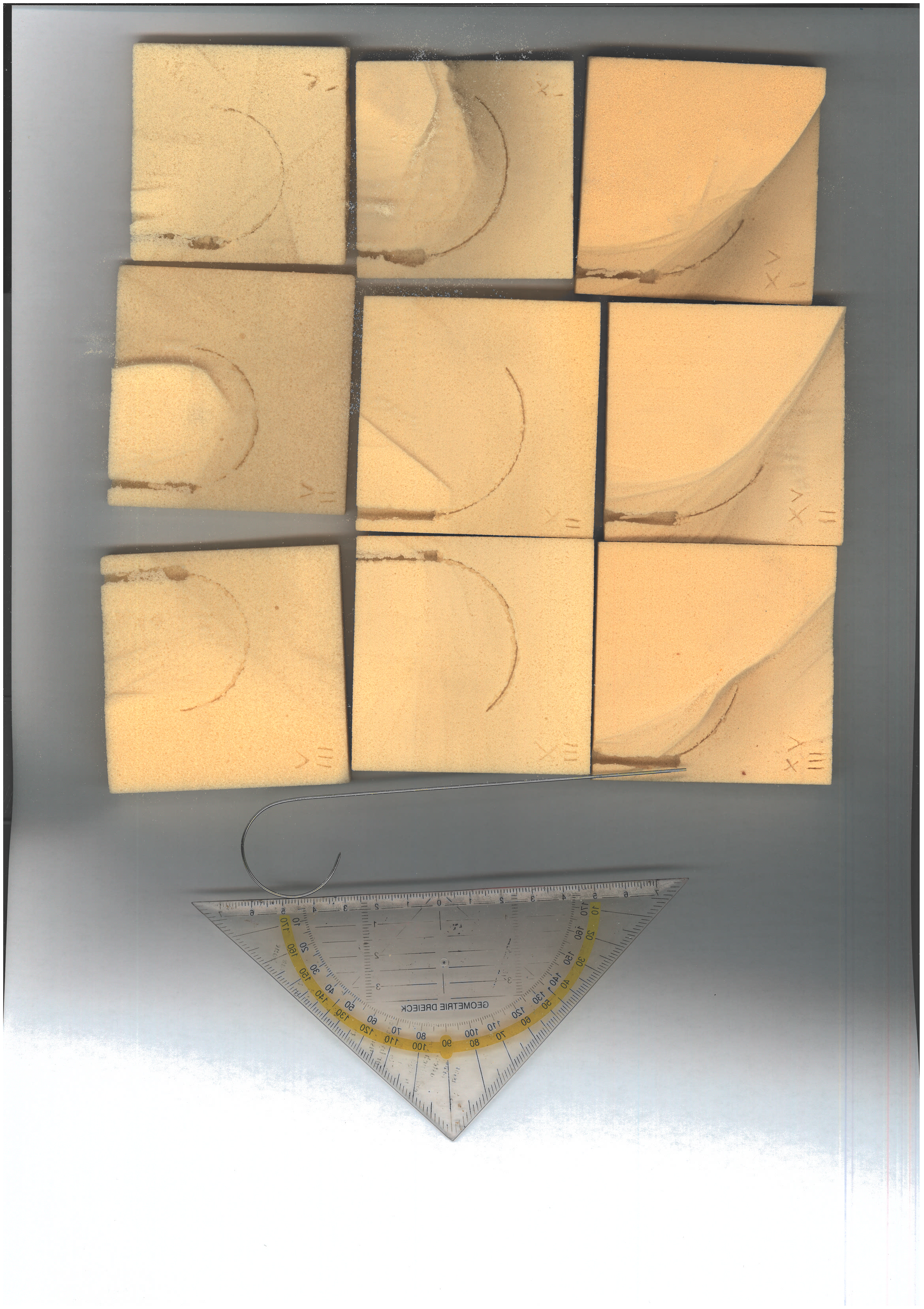

Supplement: S1 Raw image — (PNG) [file pone.0315629.s002.png]
